# Generation and evaluation of *Myostatin* knock-out rabbits and goats using CRISPR/Cas9 system

**DOI:** 10.1038/srep29855

**Published:** 2016-07-15

**Authors:** Rihong Guo, Yongjie Wan, Dan Xu, Libin Cui, Mingtian Deng, Guomin Zhang, Ruoxin Jia, Wenjun Zhou, Zhen Wang, Kaiping Deng, Mingrui Huang, Feng Wang, Yanli Zhang

**Affiliations:** 1 Jiangsu Livestock Embryo Engineering Laboratory, Nanjing Agricultural University, Nanjing 210095, PR China; 2 Department of Anesthesia, Stanford University School of Medicine, Stanford, CA 94305, USA; 3 Akeagen, Irvine, CA 92614, USA

## Abstract

*Myostatin (Mstn*) is a conserved negative regulator of skeletal muscle mass in mammals. However, whether precise disruption of *Mstn* in livestock can be achieved and safely used to improve meat productivity has not been proven. We applied CRISPR/Cas9 system to generate *Mstn* knock-out (KO) rabbits and goats and then analyzed the changes in their phenotypes to answer this question. We efficiently generated 24 *Mstn* KO rabbits out of 32 newborn infants after embryo injection with two sgRNAs targeting rabbit *Mstn*, and found that the *Mstn* KO rabbits exhibited increased birthweight and a significantly increase in the weight ratios of the quadriceps and biceps muscles to the whole body. *Mstn* KO also caused high probability of enlarged tongue phenomenon and severe health problems such as stillbirth and early stage death. Using the same method, one out of four goats was generated with edition at *Mstn* locus. The early stage growth rate of this goat outperformed the control goats. In conclusion, we efficiently generated *Mstn* KO rabbits and goats using CRISPR/Cas9 technology. However, *Mstn* KO causes severe health problems and may also have the same effects on other species. This safety issue must be studied further before applied to animal reproduction processes.

*Myostatin (Mstn*), also known as growth differentiation factor 8 (GDF-8), belongs to the TGF-beta superfamily. *Mstn* is a negative regulator of skeletal muscle mass^1^. Natural mutations that either inactivate the encoded protein or suppress its quantity cause enhanced muscling in human^2^, sheep^3^,^4^, and cattle^5^. *Mstn* KO animals, such as mice, sheep, cows and pigs with are muscular^1^,^6^,^7^,^8^. In goats, a 5 bp deletion (TTTTA) at the 5′-UTR and a 1 bp substitution (T/A) in exon 1 were reported to be associated with growth performance, such as birth body size and birthweights^9^. In rabbits, the SNPs at the 5′-UTR were reported to be associated with carcass conformation^10^. If *Mstn* KO animals can be generated without developmental and health issues, it will improve meat productivity and increase the speed of livestock breeding process.

CRISPR (Clustered Regularly Interspaced Short Palindromic Repeats)/Cas9 (CRISPR associated protein), derived from the CRISPR adaptive immune system of bacteria, can be directed to induce precise cleavage by short RNAs^11^. Compared to HR (homology recombinant), ZFN (zinc finger nuclease) and TALEN (transcription activation factor-like endonuclease), CRISPR/Cas9 is an easy-handle, highly specific, efficient, and multiplexable approach for engineering eukaryotic genomes^12^. It has been extensively and efficiently used to generate animals such as rabbits^13^, monkeys^14^, pigs^8^,^15^,^16^ and sheep^17^. Goats are an important farm animal due to their meat, milk and fur, and three *Mstn* biallelic KO goats were generated using somatic cell nuclear transfer method (SCNT)^18^. Recently using CRISPR/Cas9 system together with the zygote injection method, fifteen *Mstn* KO goats were produced out of 93 delivered lambs^19^. Taken together, the results indicated that CRISPR/Cas9 can be effectively used for precise gene targeting in farm animals.

Two studies reported that two out of four *Mstn* KO goats generated using SCNT died at birth^18^,^20^. In another study, 13 goats were stillborn and fifteen *Mstn* modified goats were born out of 93 newborn goats. However, there was no information regarding whether the 13 goats that were dead at birth contained *Mstn* modification nor the ratio of modified *Mstn* in these goats^19^. There was also no information regarding the growth rate or health conditions of the 15 *Mstn* modified goats. None of these studies presented data on the birthweight, growth performance or any health related issues. Because there is no research study of effects of natural mutations on the development, or an evaluation of the growth performance of precisely edited *Mstn* mutant animals, the generation and evaluation of the early development of *Mstn* KO goats and rabbits should be studied before *Mstn* KO technology is applied to improve meat productivity in the livestock industry. It is also critical for us to study the function of *Mstn* in goat, rabbit or other livestock animals. To initiate this evaluation, in this study, we utilized CRISPR/Cas9 system together with cytoplasm injection method to knock out *Mstn* in rabbit and goat, and then we studied the changes in the phenotypes and early stage development of *Mstn KO* rabbits and goats.

## Results and Discussion

### Efficient generation of *Mstn* KO rabbit using Cas9/sgRNA

**Figure 1:**
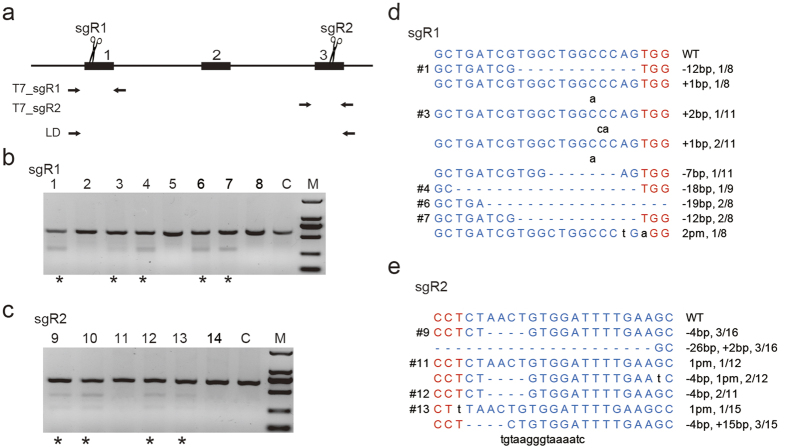
The Cas9 system mediated *Mstn* gene modifications in cultured rabbit embryos. (**a**) Schematic diagram of sgRNAs targeting the rabbit *Mstn* loci. The primer sets labeled as T7_sgR1 and T7_sgR2 were used for T7E1 cleavage assay at the sgR1 and sgR2 target sites, respectively, and primer set LD was used to detect long-range deletions of *Mstn* in rabbits developed from embryos that were injected with both sgR1 and sgR2. (**b,c**) Detection of sgR1 and sgR2 mediated cleavage in eight and six rabbit embryos respectively using the T7E1 cleavage assay. Samples with cleavaged bands were marked with asterisks. C, control embryo injected with only Cas9 mRNA; M, DNA marker. (**d,e**) DNA sequences of the marked samples in (**b,c**). TA clones from the PCR products were analyzed by DNA sequencing. The PAM sequences and targeting sites are highlighted in red and in blue, the mutated and inserted bases are indicated in lower case. WT, wild type; pm, point mutation; deletions (−), and insertions (+). N/N indicates the positive colonies out of the total colonies sequenced.

We first studied whether CRISPR/Cas9 can be used efficiently at the *Mstn* locus in rabbit embryos. After superovulation, 33.5 ± 7.86 embryos per donor rabbit were obtained for the subsequent experiments. sgR1 and sgR2 (targeted at exon 1 and exon 3 of rabbit *Mstn* respectively) were selected for embryo injection (Fig. 1a, Table S1). Twenty-six one-cell stage rabbit embryos were injected with a mixture of 200 ng/μl Cas9 mRNA and 20 ng/μl sgR1 or sgR2 (Table S3). After injection, 14 out of 26 injected embryos developed to the morula or blastocyst stages (Table S3). These embryos were collected and examined for the presence of site-specific genome modification using T7E1 cleavage assay and Sanger sequencing. The results showed that indels of various sizes (−19 bp to +2 bp in sgR1-injected embryos, and −26 bp to +15 bp in sgR2-injected embryos) occurred with an efficiency of 62.5% (5/8) for sgR1 and 66.7% (4/6) for sgR2. These results indicated that both Cas9/sgR1 and Cas9/sgR2 worked efficiently in rabbit embryos.

**Figure 2:**
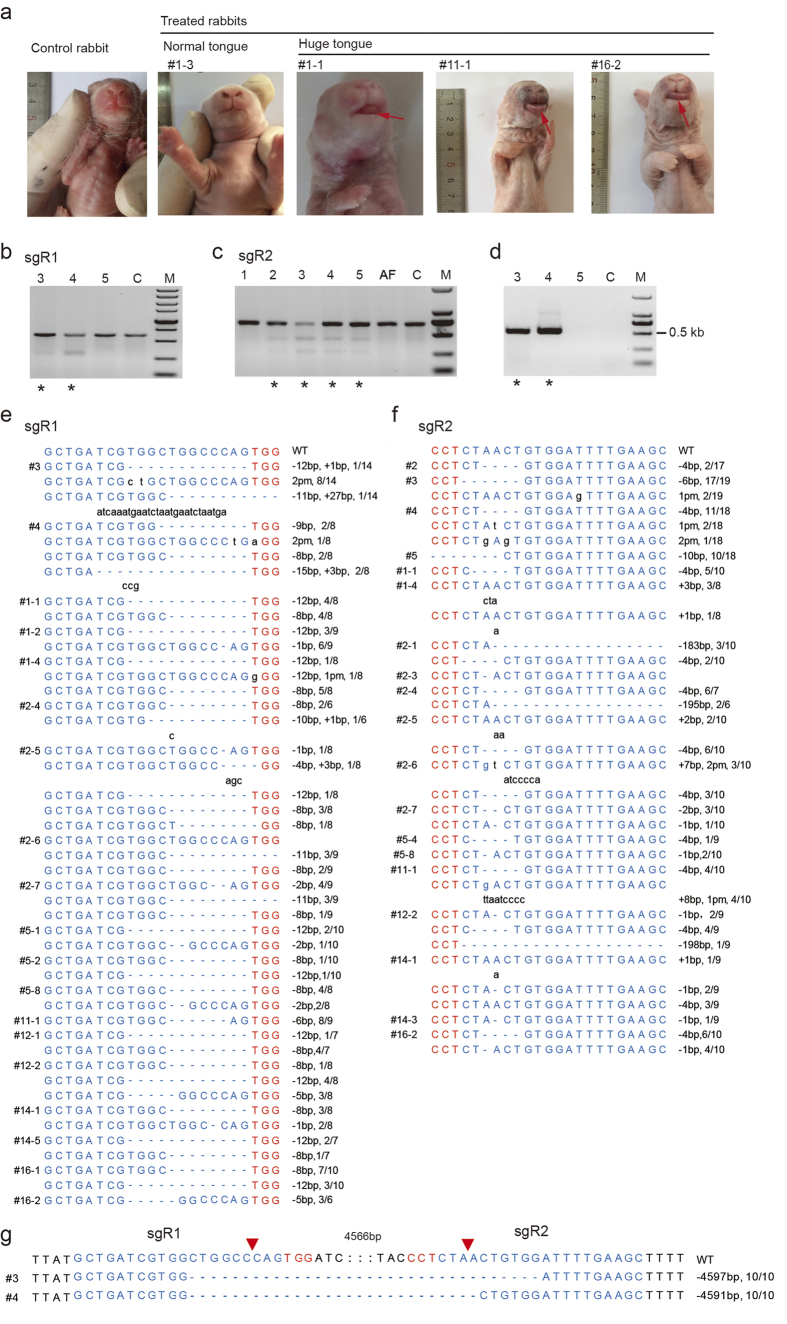
The Cas9 system mediated one-step generation of *Mstn* KO rabbits. (**a**) *Mstn* KO could resulted in enlarged tongues. The red arrows are used to highlight the enlarged tongues of infants #1-1, #11-1 and #16-2. The control rabbit was randomly selected from the 42 naturally born rabbits, the Treated rabbits were generated by embryo injection and transfer. (**b,c**) Detection of sgR1 and sgR2 mediated cleavage in newborn rabbits #1-#5 and one aborted fetus (AF) using T7E1 cleavage assay. Rabbits #1, #2 and the AF were only injected with sgR2. Rabbits #3-#5 were injected with both sgR1 and sgR2. Samples with cleavage bands were marked with asterisks. (**d**) Detection of sgR1 and sgR2 mediated long-range deletions in newborn rabbits #3-#5 using PCR. Samples with long-range deletion were marked with an asterisk. (**f–g**) DNA sequences of marked (*) samples in (**b–d**) and other samples with multi-peaks in the chromatogram after Sanger sequencing of the sgR1 and sgR2 target sites. TA clones of the PCR products were analyzed by DNA sequencing. The mutated and inserted bases are indicated in lower case. WT, wild type; pm, point mutation; deletions (−), and insertions (+). N/N indicates the positive colonies of the total colonies sequenced. The red triangles indicate the double strands break sites of sgR1 and sgR2.

After proving that the Cas9 system worked efficiently in rabbit embryos, a second batch of 383 rabbit embryos were injected as described above (Table 1). Then, a total of 315 cleaved embryos were transferred to 27 surrogate rabbits (Table 1). Twelve pregnancies were established, and one miscarriage occurred on day 12 after embryo transfer. Thirty-four rabbits were delivered from full term pregnancies (30–31 days) (Table 1). Among the newborn rabbits and the aborted fetus (AF), rabbits #1 and #2 and the AF developed from embryos injected with sgR2, and the others developed from embryos injected with both sgR1 and sgR2 (Table 1). Unexpectedly, 14 out of 34 (41.2%) infants were born with enlarged tongues (Fig. S1), among which five (#3, #4, #1-1, #11-1 and #16-2) were stillborn (Fig. 2a, Fig. S1 and Table 2). During the early infant stage, 8 more rabbits died due to weakness or were euthanized because of their poor health condition. Their tongues and other tissues were collected for further analysis. As rabbits #1 and #2 were partially eaten by their mothers due to abnormal maternal behavior, they were excluded from the analysis of the effects of *Mstn* KO on health status. The high probabilities of the huge tongue phenomenon and infant death in the *Mstn* KO infants were further discussed.

T7E1 cleavage assay was used to for the AF an infant #1-#5 (Fig. 2b,c), and direct Sanger sequencing of the PCR amplicons of the sgR1 and sgR2 sites were used for the remaining 29 infants to detect genome editing and identify the precise modifications in these sites, (Fig. S2a). The results showed that 70.6% (24/34) of the infants were edited at *Mstn* locus, 55.8% (19/34), 52.9% (18/34) and 38.2% (13/34) of the infants exhibited modifications at the sgR1 site, sgR2 site and both sgR1 and sgR2 sites, respectively (Table 2 and Table S6). We also detected indels of different sizes (−15 bp to +27 bp at the sgR1 target site, −198 bp to +8 bp at the sgR2 target site) (Fig. 2e,f). The ratio of modified DNA in each infant varies at both sites, from 20% to 100% for the sgR1 site and from 11.1% to 100% for the sgR2 site (Fig. 2e,f).

Additionally, we amplified a single product of approximately 540 bp (Fig. 2d) using primer set LD (Fig. 1a, Table S4) from the genomic DNA of rabbit #3 and #4, which indicated that a long-range deletion occurred. The long-range deletion was consistent with the fact that both the sgR1 and sgR2 sites were successfully edited in rabbit #3 and #4. However using the same primer set, we failed to amplify the wildtype (WT) band approximately of 5,000 bp and therefore didn’t know the ratio of the long-range deletion to the WT band or other small indels. Surprisingly, rabbits #3 and #4 each had only 4597 bp and 4591 bp deletion, respectively (both 10/10) (Fig. 2g). Moreover, we also noticed that both deletion types had short homology sequences between the 5′ arm of the sgR1 site and 3′ arm of the sgR2 site, for example GTGG and CTG sequences were both present in rabbits #3 and #4 (Fig. S2b). It is not clear whether 3–4 bp short sequences can influence DNA repair or mediate HR is not clear. It is recommended that the shortest template used to mediate point mutation and HR is ssODNs containing flanking sequences of at least 40 bp on each side^21^.

### Evaluation of CRISPR/Cas9 derived *Mstn* KO rabbits

**Figure 3:**
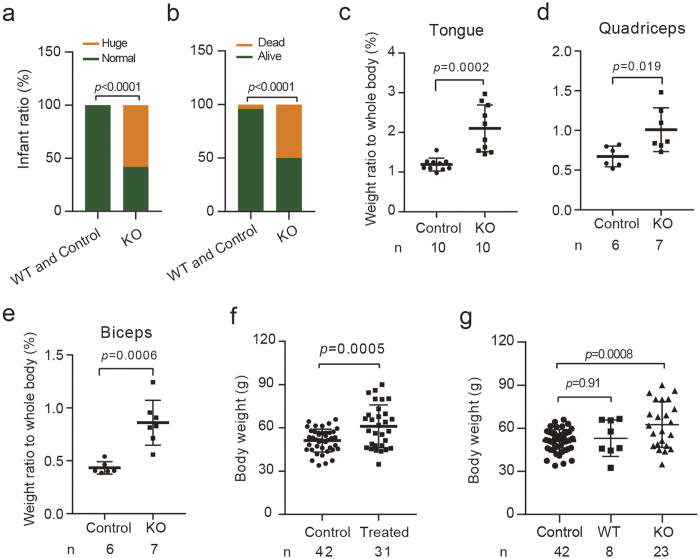
Evaluation of the effects of *Mstn* KO on tongue type, health status, birthweight, and muscularity of rabbit fetuses. (**a**,**b**) The ratios of animals with enlarged tongues (**a**) and early stage death (**b**) were significantly higher in *Mstn* KO rabbits than those of the Control and *Mstn* WT rabbits. Control (n = 42), WT (n = 8) and KO (n = 24) represent rabbits from Control group, *Mstn* WT group and *Mstn* KO group. (**c–e**) Comparison of the weight ratios of tongue (**c**) quadriceps muscle (**d**) and biceps muscle (**e**) to whole body between control rabbits and *Mstn* KO rabbits. (**f**) Comparison of the birthweight of the Treated infants with that of the Control infants. (**g**) Comparison of the birthweight of Control rabbits, *Mstn* WT rabbits and *Mstn* KO infants from the Treated group.

After genotyping all the infants, we tried to identify a potential association between genome editing and the abnormal traits such as enlarged tongue phenomenon and infant death. Surprisingly, all 14 infants with enlarged tongues were modified at the *Mstn* locus, significant high ratio (58.33%, 14/24) of abnormality compared to the control groups of 42 rabbits from 7 pregnancies (referred as the Control group hereafter, indicating naturally born infants) and 8 *Mstn* WT infant (infants from the Treated group, but neither the sgR1 site nor sgR2 sites were edited). These results clearly indicated *Mstn* KO caused the enlarged tongue phenomenon in these animals (*p* < 0.0001, Fig. 3a, Fig. S4a). Moreover, we found that *Mstn* KO also had significant effects on the infants’ health status (*p* < 0.0001) (Fig. 3b, Fig. S5b). Among the 24 *Mstn* KO rabbits, 5 (20.83%) and 7 (29.17%) infants were stillborn and dead within one week due to poor health conditions, respectively (Fig. S3b). However the incidences of stillbirth and weakness were rare in normal and *Mstn* WT infants (Fig. S4b), only 1 out of 42 control infant rabbits was stillborn and only 1 out of 8 infant from WT infant group died due to weakness.

Moreover, given that 43.8% (14 out of 32) of the infants from Treated group had enlarged tongues, we compared the weight ratio of the tongue to whole body of 10 dead infants with huge tongues (stillborn or dead within 1 week after birth due to weakness) with that of 10 random selected infants from the Control group. Consistent with the appearance, the tongue/body weight ratio had increased 78.4% in infants with enlarged tongues compared with the Control group (2.10 ± 0.59% vs 1.19 ± 0.16%, *p* = 0.002) (Fig. 3c). Similar to observations from other *Mstn* KO animals such as sheep^6^, Cows^6^ and pigs^7^,^8^, *Mstn* KO in rabbits also caused muscularity. The weight ratios of quadriceps and biceps muscles in rabbits with enlarged tongue increased by 50.12% (1.01 ± 0.27% vs 0.67 ± 0.13%, *p* = 0.019) and 98.3% (0.86 ± 0.21% vs 0.43 ± 0.059%, *p* = 0.0006) respectively compared to the normal rabbits (Fig. 3d,e).

Next we compared the birthweight of those 32 newborn rabbit from Treated group with the infants from Control group. As expected, the birthweight of Treated group was significantly higher than that of the Control group (61.1 ± 14.87 g vs 51.24 ± 7.97 g, *p* = 0.0011) (Fig. 3f). This result indicated that *Mstn* KO could enhance the birthweight of newborn rabbits. To precisely evaluate the effects of *Mstn* KO on birthweight, the treated infants were divided into the *Mstn* WT group and the *Mstn* KO group. The birthweight of the *Mstn* WT group was almost the same as that of the Control group (51.6 ± 12.12 g vs 53.09 ± 12.63 g, *p* = 0.91) (Fig. 3g), whereas the edited group was 22.2% heavier than the control rabbits (62.63 ± 18.94 g vs 51.24 ± 7.97 g, *p* = 0.008). Notalbly, the *Mstn* editing efficiencies of four rabbits in *Mstn* KO group were less than 30% (11.1%, 11.1%, 20% and 30%), and the classification method described above may underestimate the influence of *Mstn* KO on birthweight. This speculation was confirmed by the larger difference between *Mstn* KO efficiency ≤30% group and >30% group (51.08 ± 10.67 g vs 64.9 ± 13.88 g, *p* = 0.0014) (Fig. S3). Additionally, stillborn infant #11-1 was fully KO at *Mstn* locus, its birthweight was approximately 2 times higher than the mean birthweight of the *Mstn* KO group (115.3 g vs 62.63 ± 18.94 g), and was excluded from the birthweight analysis.

**Figure 4:**
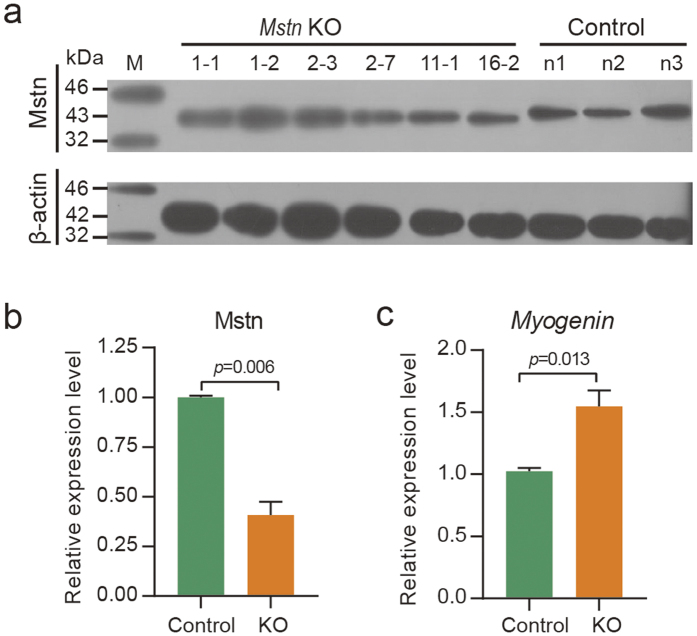
Detection of Mstn and *Myogenin* expression level in skeletal muscle. (**a**) Western blotting analysis of Mstn in skeletal muscle of infant rabbits. (**b**) Quantification of the relative Mstn protein level by gradation analyses. (**c**) The relative expression of *Myogenin* in skeletal muscle was determined using qRT-PCR. The 6 *Mstn* KO infants tested were #1-1, #1-2, #2-3, #2-7, #11-1 and #16-2, and the 3 control rabbits were n1–n3.

Consistent with the genome editing results, the Mstn protein level in KO rabbits were significantly reduced compared to the control rabbits (Fig. 4a,b). This result could be explained by the presence of frameshift indels in the KO rabbits. There were also indels that didn’t result in frameshift shift, but instead resulted in deletion of several amino acids. These mutations may not be detected by Western blotting analysis, but they may also inactivate Mstn protein. We also detected the expression of *myogenin*, a downstream myogenic regulatory factor of *Mstn* that affects muscularity^8^. The result showed that the decrease in the quantity and the inactivation of Mstn protein expression upregulated *myogenin* (Fig. 4c).

### Generation of *Mstn* KO Goats

**Figure 5:**
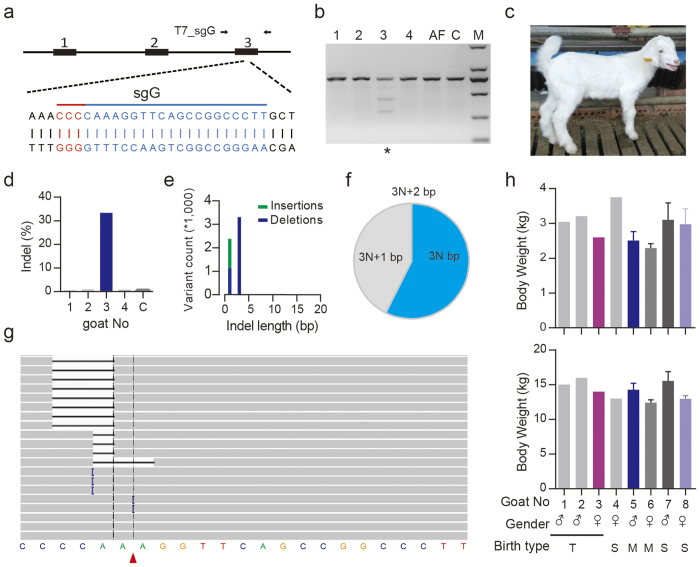
The Cas9 system mediated generation of *Mstn* KO goat. (**a**) Schematic diagram of the sgG targeting goat *Mstn* loci. The PAM sequences and targeting sites are highlighted in red and blue, respectively. The primer set T7_sgG was used for T7E1 cleavage assay at the sgG target site. (**b**) Detection of sgG:Cas9-mediated cleavage at *Mstn* loci of the 5 newborn goats by T7E1 cleavage assay. C, control goat; AF, aborted fetus. (**c**) Photo of the genome edited goat #3. (**d**) Indel frequency at the *Mstn* locus in ear genomic DNA. (**e**) Distribution of the indel length. (**f**) Distribution of indel frame phase calculated as the length of indels modulus 3. For example, 1-, 4- and 7-base-pair indels are 3N + 1, 2-, 5- and 8-base-pair indels are 3N + 2 and 3-, 6- and 9-base-pair indels are 3N. The pie chart shows the percentage of each class of indel. (**g**) Representative views of indels in ear genomic DNA of goat #3 using the Integrative Genomics Viewer. The black or purple bars indicate deletions or insertions, respectively. (**h**) The birthweight (BW0) (upper graph) and 4 month body weight (BW4) (lower graph) of four live newborn goats and control goat herds. Goat #1 (♂), #2 (♂), and #3 (♀) were from a triplet pregnancy and goat #4 (♀) was from a single pregnancy, control goat herds 5 (n = 5, ♂) and 6 (n = 4, ♀) were from multiple pregnancies, and control goat herds 7 (n = 4, ♂) and 8 (n = 4, ♀) were from single pregnancies. T, M, S represent triplet, multiple (triple and double), and single birth types, respectively.

Next, we generated *Mstn* KO goats using Cas9 system and single cell embryo injection to determine whether the abnormality was species-specific. After superovulation, 21 one-cell stage embryos were harvested from one donor goat. sgG targeted exon 3 of goat *Mstn* was selected (Fig. 5a) for the subsequent experiments. Based on the result from the rabbit study that complete *Mstn* KO caused health related issues, we used a reduced amount of Cas9 mRNA and sgRNA in this study. Therefore, 50 ng/μl Cas9 mRNA and 10 ng/μl sgG were used for the injections (Table 1). Eighteen cleaved embryos were transferred to 5 recipient goats. Thirty days after embryo transfer, 3 pregnancies (3/5, 60%) were established as indicated by ultrasonography detection (Table 1). Of the 3 pregnant goats, one miscarried on day 120 after embryo transfer; the other two underwent full gestation period (145 days) and delivered 4 healthy infant goats. Goats #1-#3 were born from a triplet pregnancy, and goat #4 was born from a single pregnancy.

Genomic DNA was extracted from the ear tissue of an abortive fetus (AF) and infant goats for T7E1 cleavage assay. One out of five goats had modification at the sgG site (Fig. 5b,c). As indicated above, the reduced Cas9 mRNA and sgRNA concentrations may reduce genome editing efficiency. To fully explore the indels, sequence around sgG target site was amplified for deep sequencing. The data indicated that 33% of the deep sequencing reads were modified (Fig. 5d), the indels were mainly 3 bp deletions and ±1 bp indels (Fig. 5e,f,g, Fig. S5a). The 3 bp deletion (–CCA) resulted in deletion of a lysine and a proline to glutamine substitution, and another different 3 bp deletion (-AAA) resulted in deletion of a lysine (Fig. S5b,c). The proline is conserved in different species (Fig. S5d) and the lysine is also conserved in goat and sheep and substituted with another basic amino acid, arginine, in other animals (Fig. S5d). Moreover, the ±1bp indels should lead to frameshift mutation and premature transcription termination (Fig. S5). Considering that the C-terminal region is important for Mstn function, the amino acid deletions and substitutions in our study may lead to a loss of function.

### Evaluation of the *Mstn* KO goats generated using CRISPR/Cas9

The birthweight (BW0) of goat #3 (♀) was the lowest among the four newborn goats (Fig. 5h, upper graph). It can be interpreted by birth type and gender effect that larger litter size is correlated with lower BW0 and BW0 of male kids is higher than that of female kids. Goat #4 (♀) from a single pregnancy was heavier than the other goats from the triplet pregnancy. Goat #3 was the only female from the triplet pregnancy, and it had the lowest BW0. Four months later, the weight (BW4) of goat #3 was still lower than her male sibs but she was heavier than the other female goat #4 (Fig. 5g, lower graph). The tendency of the slightly higher growth rate in goat #3 may be the effect of the 33% *Mstn* KO. The body weights of the four *Mstn* targeted goats (BW0 and BW4) were higher than the weights of normal goats in the control group (goat herds #5-#8). BW0 and BW4 of goat #3 were comparable to those of the control goats from multiple pregnancies. These results could be explained by two reasons: first, the recipient goats received better nutrition during the gestation. Second, after delivery, four *Mstn* KO goats were raised by a single mother individually while the control goats from a multiple pregnancy were raised by a female goat. Additionally, the newborn goats generated from CRISPR/Cas9 construct only were all healthy. It indicated that the CRISPR/Cas9 construct exhibited litter toxicity and didn’t affect newborn development. This observation was consistent with the results in a rat study^22^.

In our previous study using the HR and SCNT methods, four out of 5 full-term *Mstn* KO goats were stillborn, the only kid that survived for more than 8 months was born with an enlarged tongue (data not published). In addition, two other studies also reported a low survival rate after *Mstn* gene editing^6^,^20^. Among 32 *Mstn* KO pigs generated using TALEN and SCNT methods, only 13 pigs survived for more than 8 months and only 1 out 2 pigs were healthy^20^. Additionaly, 8 *Mstn* KO pigs were generated using CRISPR/Cas9 combined with SCNT, 2 (25%) piglets were born with enlarged tongues, and none of them survived for more than one week^6^. Although double-muscled (DM) calves were more likely to die at the perinatal stage or be born with enlarged tongues^22^, such high ratio of enlarged tongue phenomenon and infant death had never been reported in *Mstn* KO animals and were previously ascribed as the side effects of *Mstn* KO before. In the past several decades, low gene editing efficiency, abnormal fetus development and high fetal mortality were considered as consequences of limitations of SCNT technology in cloned animals, there was no attempt to link the abnormalities to a direct effect of *Mstn* KO on the phenotype of those gene edited animals. However using CRISPR/Cas9 technology and single cell embryo injection, we (Fig. S4b and Table 1) and other groups demonstrated that single cell embryo injection method and CRISPR/Cas9 technology alone didn’t affect the health conditions of animals^23^,^24^. Most importantly, for the first time we presented direct evidence that *Mstn* KO caused abnormalities in gene edited animals, which suggested that *Mstn* KO may not be an ideal way to improve the muscle mass in rabbits, and also in animals, such as pigs and goats.

### Detection of mutations in different somatic tissues

Early reports demonstrated that gene mutations in mouse and rats generated by CRISPR/Cas9 method are heritable in mouse and rats^23^,^24^. Recently it was confirmed that mutations can be efficiently transmitted into gonads and germline in single cell level^25^. In this study, we also analyzed the genotype of different somatic tissues from rabbits #3-#5 at the sgR2 site and goat #3 at the sgG site using T7E1 assay. As expected, in rabbits #3-#5 mutations had been transmitted to tongue and muscle as well as other somatic tissues (Fig. S6a). In goat #3, placenta, umbilical cord, blood and ear tissue also had inherited the mutations (Fig. S6b). The indel type (Fig. S6C) and frequency (Fig. S6d) in the blood genomic DNA were almost the same as those in the ear genomic DNA. It can be expected that the mutations could be transmitted to the next generation through the germline in goat, and rabbits.

### Off-target analysis

Off-target effects are a major concern for the CRISPR/Cas9 system^26^. We screened a total of 27 potential off-target sites (OTS), including 8 OTS for sgR1, 9 OTS for sgR2, and 10 OTS for sgG to test whether off-target effects occurred and exclude the possibility that the off-target effects occurred in key genes required for development, subsequently causing the death of the newborn rabbits (Table S2). T7E1 cleavage assay showed that there were no detectable off-target effects in our study (Fig. S7), which indicated that the gRNAs used in our study is specific.

In conclusion, we efficiently generated *Mstn* KO rabbits and goat, as well as found that *Mstn* KO improved the birthweight and growth rate of newborns and is a powerful tool to improve domestic animal breeding and meat production. However, *Mstn* KO may cause severe health problems. Using the homogeneous *Mstn* KO offspring, safety issues need to be addressed in future studies before the technology can be utilized in agricultural area.

## Methods

### Animals

Two- to three- year-old healthy goats were selected and housed at Haimen Goat R&D Center in Jiangsu. Six-month-old to one-year-old healthy rabbits were chosen and housed at Jiangsu Livestock Embryo Centre, Nanjing Agricultural University. All protocols involving the use of animals were performed in accordance with the approved Guidelines for Animal Experiments of Nanjing Agricultural University and were approved by the Animal Care and Use Committee of Nanjing Agricultural University (Approval ID: SYXK2011-0036).

### Preparation and injection of one-cell embryos

Rabbits and goats were treated using the superovulation protocol indicated in Table S5. Donor rabbits, average weight 2.82 ± 0.23 kg, were treated with a total of 75 IU FSH twice daily in decreasing doses over 3 days (20/20, 10/10 and 7.5/7.5 IU), followed by 100 IU of hCG 12 h later and were mated immediately. Twenty hours after mating, fertilized embryos were flushed from the oviducts. One-cell stage embryos were microinjected with a mixture of Cas9 mRNA and sgRNAs (Table 1 and Table S3). Microinjections were performed in the cytoplasm of zygotes using a Nikon microinjection system (NT-88-V3, Nikon, Japan) under standard conditions. The cleaved embryos were either cultured in M2 medium (Sigma-Aldrich, M5910) for T7E1 cleavage assay, or transferred into recipients that had been treated with 100 IU GnRH and given manual vulval stimulation 24 h before transfer. All the hormone or analogues used in this study were provided by Sansheng Pharmaceutics (Ningbo, China).

Progesterone sponges were intravaginally implanted in goats for 11 days, followed by the administration of 100 IU PG at the time of sponge removal (Table S5). The donors received a total of 200 IU FSH twice daily in a decreasing dose over 3 days (50/50, 25/25 and 25/25 IU) starting 48 h before sponge removal. Then donors were mated at 36 h and 48 h after sponge removal. The recipients received 100 IU PMSG 24 h prior to sponge removal. One-cell stage embryos were flushed from the donor oviducts 72 h after sponge removal. Harvested one-cell stage goat embryos were then injected with a mixture of 50 ng/ul Cas9 mRNA and 10  g/μl sgG (Table 1). Injected zygotes were then cultured in M2 media containing 10% FBS (Gibco, 26140) at 37 °C in 5% CO_2_. A total of 18 cleaved embryos at two-cell to blastocyst stage were transferred into five estrous-synchronized recipient goats. Early pregnancy was confirmed by ultrasonography 28–30 days after embryo transfer.

### sgRNAs design

sgRNAs targeted to goat and rabbit *Mstn* (Figs 1a and 5a) were designed and inserted to pX330 plasmid as described by Ran^21^. Twenty base pair sequence directly upstream of any 5′-NGG were identified using CRISPR Design tool (http://crispr.mit.edu/). The sgRNAs for rabbits were scored using this tool. The potential OTs of sgRNAs for goat were computationally predicted using Cas-Offinder^27^ according to the goat genome assembly v1.0^28^. sgG for goat, sgR1 and sgR2 for rabbits were selected because they displayed fewer potential off-target sites (Table S1). The oligoes for each sgRNA (Table S4) were annealed and cloned into the pX330 plasmid, which has a Cas9 expression cassette and sgRNA cassette.

### *In vitro* transcription

The *in vitro* transcription templates for Cas9 and sgRNAs were amplified using the T7 promotor-appended primers listed in Table S4, and were gel-purified using QiaQuick Spin Column (Qiagen, Germany). The Cas9 template was subjected to T7 Ultra Kit (Ambion, AM1345) and the sgRNA templates were transcribed using MEGAshortscript Kit (Ambion, AM1354) *in vitro*. All of the Cas9 mRNA and sgRNAs were purified using the MEGAclear Kit (Ambion, AM1908).

### T7E1 cleavage assay and Sanger sequencing of the PCR amplicons

Ear genomic DNA was extracted using a DNA extraction kit (Tiangen, DP348), and genomic DNA was extracted from rabbit embryos and amplified using the REPL1-g Single Cell Kit (Qiagen, 150343) following the manufacturer’s protocol. The genomic regions surrounding each CRISPR target site were amplified using PCR using primers listed in Table S4, then purified using QiaQuick Spin Column (Qiagen, 28704) following manufacturer’s protocol. A total of 200 ng of the purified PCR product was mixed with NEB Buffer 2, and subjected to the reannealing process to enable heteroduplex formation: 95 °C for 10 min, 95 °C to 85 °C ramping at −2 °C/s, 85 °C to 25 °C at −0.25 °C /s, and 25 °C hold for 1 min. After reannealing, products were treated with 0.5 μl T7E1 (NEB, M0302L) for 30 min, and resolved on 2% agarose gel. The PCR products with mutations detected by T7E1 cleavage assay or Sanger sequencing were then sub-cloned into pMD-19T vector (Takara, D103A). For each sample, 8 to 25 random colonies were used for sequencing.

### Western blotting analysis

Mstn protein in rabbit skeletal muscle was detected by Western bloting analysis. The proteins were prepared using radioimmunoprecipitation assay (RIPA) buffer (Beyotime, Haimen, China) and the concentrations were determined using the bicinchoninic acid (BCA) assay (Ding Guo, Nanjing, China). Then, denaturation was performed in sodium dodecyl sulfate (SDS) gel-loading buffer at 100 °C for 10 min. Total protein (40 μg per sample) were separated by 12% SDS-polyacrylamide gel electrophoresis (SDS-PAGE) and electrotransferred to polyvinylidene fluoride (PVDF) membranes (Millipore; Billerica, USA). After incubation in blocking buffer (5% BSA in Tris-buffered saline containing 0.1% Tween 20) for 1 h at RT, the membrane was incubated overnight at 4 °C with a mouse anti-Mstn primary antibody (Sigma, SAB5300419, 1:1,000 dilution) and mouse anti-β-actin primary antibody (Santa Cruz Biotechnology, SC-47778, 1:1,500 dilution). After washing, the membrane was incubated with a goat anti-mouse IgG (H + L) secondary antibody (Thermo Pierce, 31160, 1:5,000 dilution) for 1 h at RT. After washing, the signal was detected using an ECL western blotting detection system (Fujifilm, Tokyo, Japan), and the chemiluminescence intensity of each protein band was quantified using ImageJ software.

### Gene expression analysis

Total RNA was extracted from the skeletal muscle of the *Mstn* KO rabbits and control rabbits using TRIzol reagent (Invitrogen, 15596026), and the RNA concentration was quantified using a NanoDrop spectrophotometer. First-strand cDNAs were generated through reverse transcription using 1 ug of total RNA and oligo-dT primers. The sequences and GeneBank accession numbers of the primers sets used to ampllify of the target genes are presented in Table S4. The qPCR assessment was performed using an ABI 7500 Real-Time PCR System (Applied BioSystems Carlsbad, CA) and fluorescence was detected using SYBR Green (Roche, Germany) in a reaction volume of 20 μl. The quantity of each measured cDNA sample was normalized to the reference gene *β-actin*. The relative expression levels of the target gene were quantified using the ΔΔCT method. For ease of comparison, the average expression level of each gene from the control rabbits was set at 1.00.

### Deep sequencing of the goat *Mstn* locus

The genomic region surrounding the sgG target site in goat #3 was amplified by PCR using Herculase II high-fidelity polymerase (Agilent, 600675) and then purified. Libraries were made from 20 ng of the PCR products then approximately 300 bp were amplified by PCR with primers that contain indexes. Next the products were sequenced on Illumina MiSeq machines. Data was processed according to standard Illumina sequencing analysis procedures. Briefly, reads were mapped to the PCR amplicons as reference sequences using Burrows–Wheeler Aligner with custom scripts33. Insertions and deletions were crosschecked against reference using VarScan2. The indel phase was calculated as the length of insertions or deletions modulus 3.

### Data analysis

The genome editing efficiencies for sgR1 (*p*1), sgR2 (*p*2) and sgG were the ratios of the the positive TA-clones to total clones sequenced, and the genome editing efficiencies for each rabbit infant were calculated as *p* = (1–(1–*p*1) * (1–*p*2)) * 100. Two-sided unpaired Student’s t-tests were performed to compare the difference of birthweight, weight ratios of the tongue, quadriceps muscle and biceps muscle to the whole body between *Mstn* KO rabbits and control rabbits. One-way ANOVA was used to compare the birthweight of different genotypes of *Mstn*. The error bars represent the standard deviation (SD).

## Additional Information

**How to cite this article**: Guo, R. *et al*. Generation and evaluation of *Myostatin* Knock-out Rabbits and Goat Using CRISPR/Cas9 system. *Sci. Rep.* **6**, 29855; doi: 10.1038/srep29855 (2016).

## Supplementary Material

Supplementary Information

## Figures and Tables

**Table 1:** Summary of embryo microinjection with Cas9 mRNA and sgRNA.

**Species**	**Cas9 mRNA**	**sgRNA (concentration)**	**Embryos transferred/Embryos injected**	**Pregnancies/Recipients**	**Fetus/Stillborn**
Rabbit	200 ng/μl	sgR2 (20 ng/μl)	85/65	2/6	2/0
Rabbit	200 ng/μl	sgR1 + sgR2 (20 + 20 ng/μl)	80/56	3/6	3/2
Rabbit	100 ng/μl	sgR1 + sgR2 (20 + 20 ng/μl)	218/194	7/15	29/3
Goat	50 ng/μl	sgG (10 ng/μl)	21/18	3/6	4/0

**Table 2:** Summary of the effects of *Mstn* KO on tongue type and health status of infants.

**Group**	**Genome editing**	**Tongue type**		**Health status**			**Total**
		**Normal**	**Enlarged**	**Stillborn**	**Weak**	**Alive**	
Treated (n = 32)	KO	10	14	5	7	12	24
	WT	8	0	0	1	7	8
Control (n = 42)	KO	0	0	0	0	0	0
	WT	42	0	0	41	41	42
Total (n = 74)	KO	10	14	5	7	12	24
	WT	50	0			48	
