# Supplementary material for: Generation and evaluation of Myostatin knock-out rabbits and goats using CRISPR/Cas9 system
Source: Sci Rep. 2016 Jul 15;6:29855. doi: 10.1038/srep29855 (PMC4945924; doi:10.1038/srep29855)
Supplement: Supplementary Information [file srep29855-s1.pdf]

Supplementary information

Generation and evaluation of *Myostatin* Knock-out Rabbits and Goat Using CRISPR/Cas9 system

Rihong Guo[1+], Yongjie Wan[1+], Dan Xu[2], Libin Cui[3], Mingtian Deng[1], Guomin Zhang[1], Ruoxing Jia[1], Wenjun Zhou[1], Zhen Wang[1], Kaiping Deng[1], Mingrui Huang[1], Feng Wang[1*] & Yanli Zhang[1*]

[1] Jiangsu Livestock Embryo Engineering Laboratory, Nanjing Agricultural University, Nanjing, PR China, [2] Department of Anesthesia, Stanford University School of Medicine, Stanford, CA 94305, USA, [3] Akeagen, Irvine, CA 92614, USA.

* Corresponding authors: Feng Wang and Yanli Zhang. Jiangsu Livestock Embryo Engineering Laboratory, Nanjing Agricultural University, Nanjing 210095, China. Tel: +86 25 84395381; fax: +86 25 84395314; E-mail: Feng Wang, caeet@njau.edu.cn; Yanli Zhan, zhangyanli@njau.edu.cn.

+ These authors contributed equally to this work.

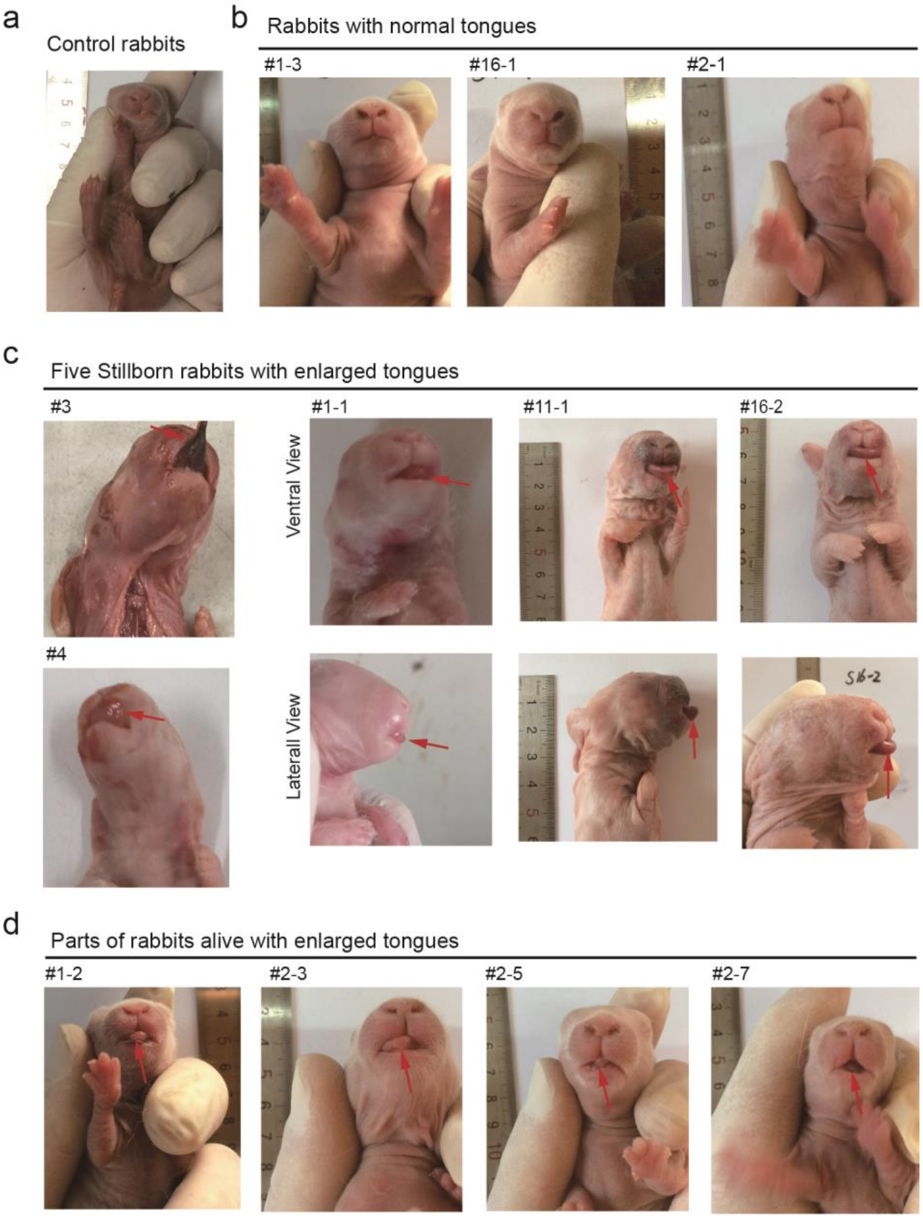

Figure S1 | Typical tongue photos of newborn rabbits. **a**, The control rabbits were born with normal tongues. **b**, *Mstn*-WT infants (shown here is #1-3) and parts of *Mstn*-KO infants (shown here are #16-1 and #2-1) were with normal tongues. **c**, Five *Mstn*-KO infants (#3, #4, #1-1, #11-1, #16-2) were stillborn and with enlarged tongues. Both ventral views and lateral views were presented for infants #1-1, #11-1 and #16-2. **d**, Parts of the *Mstn*-KO infants (shown here are #1-2, #2-3, #2-5, #2-7) were live-born and with enlarged tongue. Read arrows were used to highlight the enlarged tongues.

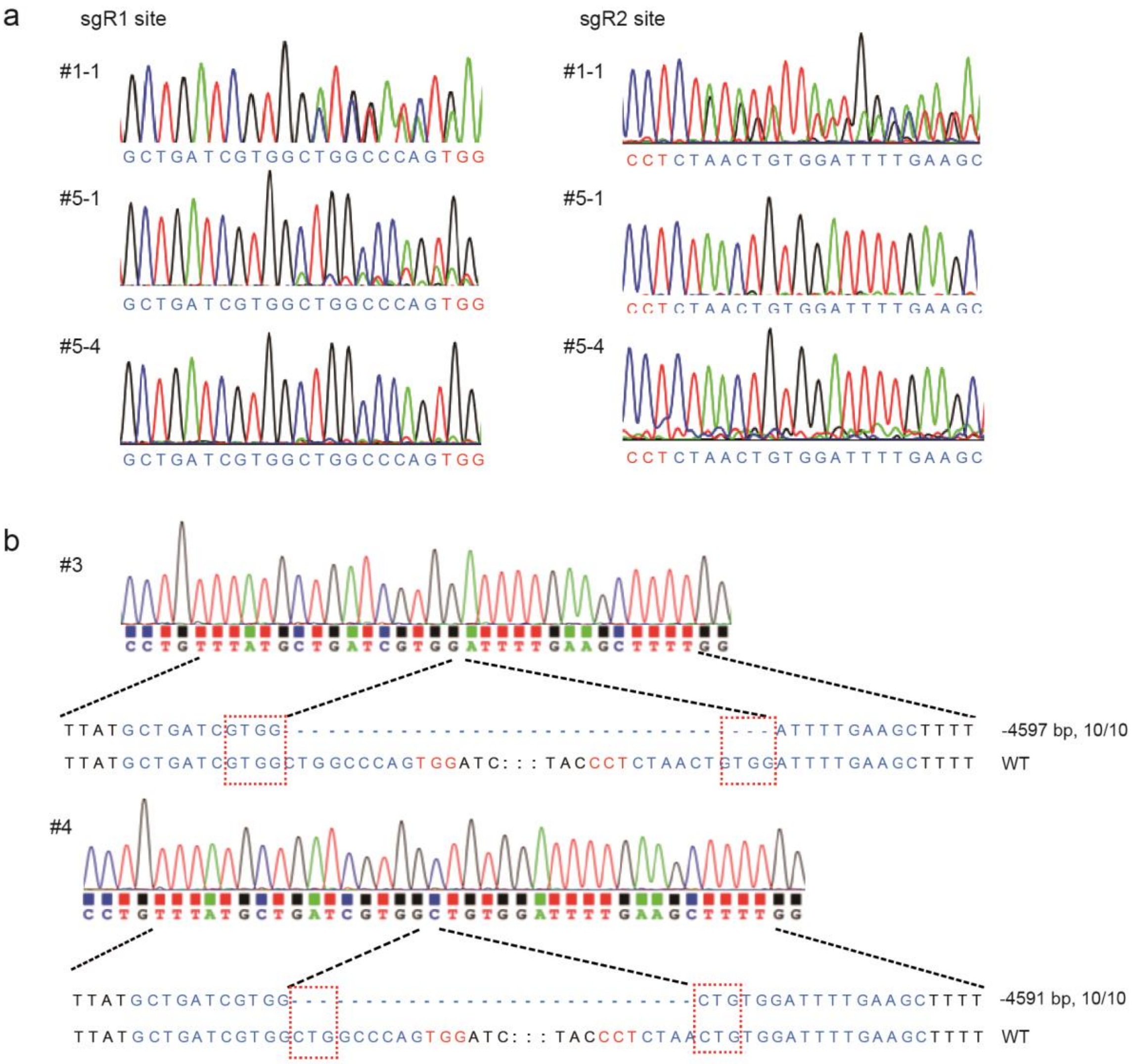

Figure S2 | Typical chromatographs of sgR1 and sgR2 target sites and long-range deletions of #3 and #4. a, Double peaks in chromatographs of #1-1, #5-1, #16-2. Both sgR1 and sgR2 sites were edited with high efficiencies in #1-1, sgR1 site and sgR2 site edited with low efficiencies respectively in #5-1 (30%) and #5-4 (11.1%) could also be detected by direct Sanger sequencing of PCR amplicons. **b**, Sequencing results of -4597 bp deletion in rabbit #3 and -4591 bp deletion in rabbit #4. The short homology sequences GTGG of #3 and CTG of #4 were boxed in red rectangles.

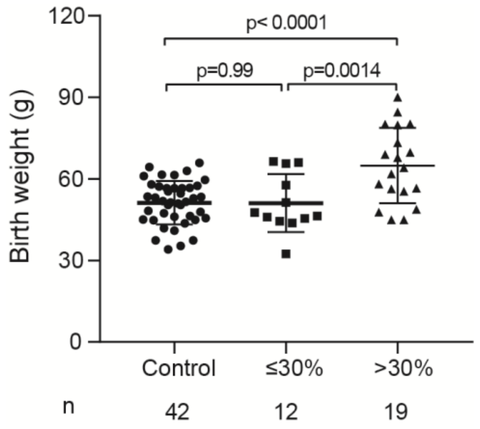

Figure S3 | Birth weight of the newborn rabbits. The 42 control rabbits were naturally born from 7 litters, the Treated rabbits were divided into genome editing efficiency ≤30% group (*Mstn* WT infants included) and >30% group.

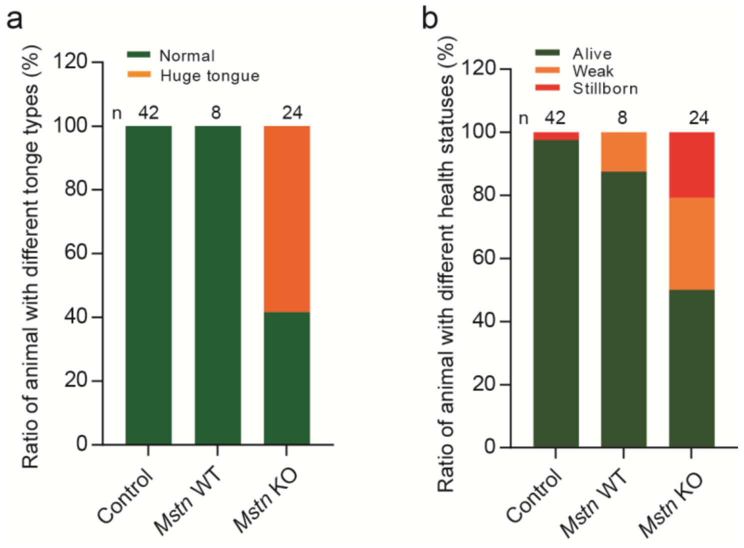

Figure S4 | *Mstn* KO affects tongue type and health status. Only the *Mstn* KO rabbits had huge tongues (**a**), and the stillbirth rate and weak animal both were all higher in *Mstn* KO rabbits (**b**). The 42 control rabbits were from 7 naturally born litters, *Mstn* WT means infants from the treated group though no genome editing was found at *Mstn* locus, *Mstn* KO means infants were successfully KO at *Mstn*-locus.

a

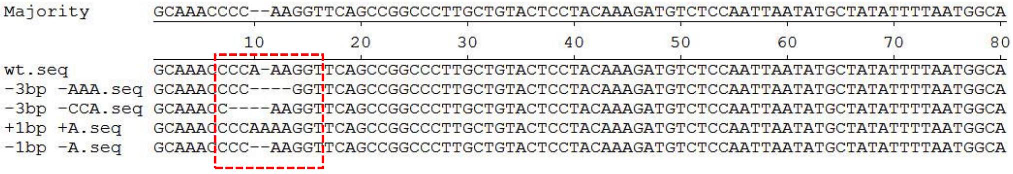

b

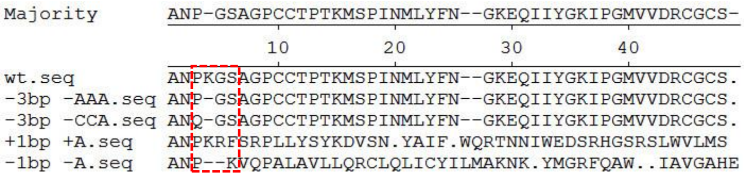

c

Indel type	Protein mutation
-3 bp CCA	Lysine deletion, proline substituted by glutamine
-3 bp AAA	Lysine deletion
-1 bp A	Open reading frame disruption
+1 bp A	Open reading frame disruption

d

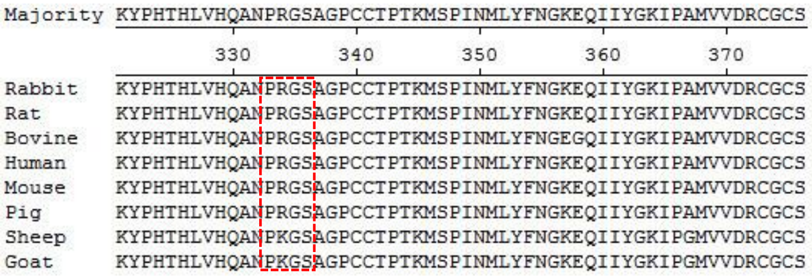

Figure S5 | Four kinds of mutations at *Mstn* locus of goat #3 and their correspondence protein mutation. a, Multi-alignment of four mainly mutations. **b**, Correspondence protein mutations of the four mutations in **a**. **c**, Summary of the protein mutations. **d**, Multi-alignment of protein sequences from 8 mammal animals. The mutated sites are in red boxes in **a, b, d**.

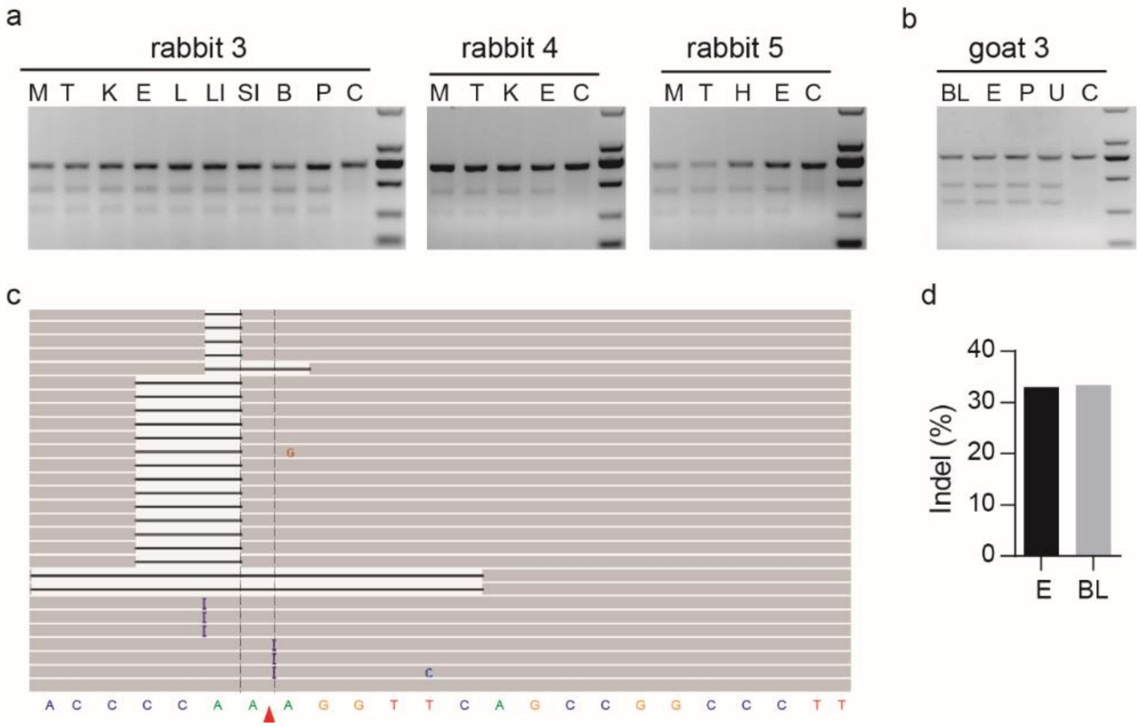

Figure S6 | Cas9 mediated modifications in somatic tissues of rabbits and goat. a, Detection of modification by T7E1 cleavage assay in somatic tissues of rabbits #3~5 at sgR2 site. M, skeletal muscle; T, tongue; H, heart; K, kidney; L, lung; LI, liver; B, brain; SI, small intestine; P, placenta; T, tail; C, wild-type control. **b**, Detection of modification by T7E1 cleavage assay in somatic tissues of goat #3 at sgG site. BL, blood; E, ear; P, placenta; U, umbilical cord; C, control. **c,** Representative views of indels in blood genomic of goat #3 using the Integrative Genomics Viewer. **d**, Indel frequences at *Mstn* locus in ear (E) and blood (BL) genomic DNA.

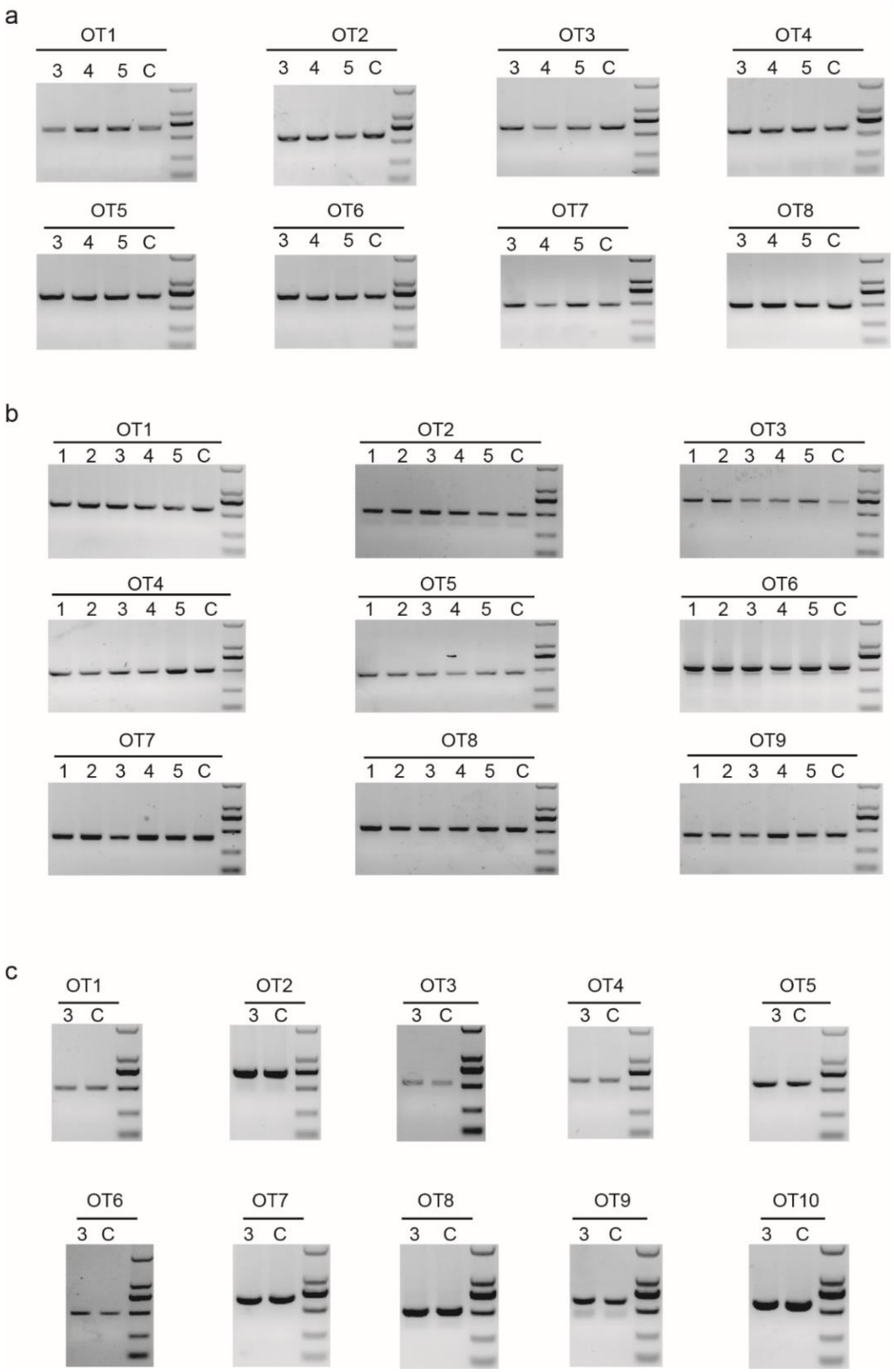

Figure S7 | Detection of 27 potential OTs using T7E1 cleavage assay. a, T7E1 cleavage assay of 8 potential OTs of sgR1. **b,** T7E1 cleavage assay of 9 potential OTs of sgR2. **c,** T7E1 cleavage assay of 10 potential OTs of sgG.

Table S1 | Details of gRNAs for goat and rabbit.

Species	Gene target	gRNA	Sequence (5'→3')
Rabbit	*Mstn* Exon1	sgR1	GCTGATCGTGGCTGGCCCAGTGG
	Mstn Exon3	sgR2	GCTTCAAAATCCACAGTTAGAGG
Goat	*Mstn* Exon3	sgG	gAAGGGCCGGCTGAACCTTTGGGG

Table S2 | 27 Potential off-target sites of sgG, sgR1 and sgR2.

gRNA	OTs	Sequence (5'→3')	Mismatches	Chromosome
sgG	OT1	AGGGGCTGGCTGAATCTTTGAGG	3MMs [2:7:15]	chr1
	OT2	AAGGGCTGGCTGTACCATTGAGG	3MMs [7:13:17]	chr14
	OT3	AAGGGCCTGTTGAACATTTGAGG	3MMs [8:10:15]	chr14
	OT4	AAAGGCCGGCTGCGCCTTTGAGG	3MMs [3:13:14]	chr23
	OT5	CAGGGCTGGCTGAACATTTGTGG	3MMs [1:7:16]	chr7
	OT6	ATGGGACAGCTGAACCTTGGAGG	4MMs [2:6:8:19]	chr16
	OT7	CAGGGCTGGCTGAGCCTTCGTGG	4MMs [1:7:14:19]	chr2
	OT8	TGGGTCCGGCTGAACCTGTGTGG	4MMs [1:2::4:17]	chr2
	OT9	TATGGACGGCTGAACCTTGGAGG	4MMs [1:3:6:19]	chr22
	OT10	GAGGGAGGGCTGAACCTTAGGGG	4MMs [1:6:7:19]	chr29
sgR1	OT1	GCTGATGGTGGCTGGCCCTGCAG	2MMs [7:19]	chr14
	OT2	GATGCCCGTGGCTGGCCCAGGAG	3MMs [2:5:6]	chr13
	OT3	GCTGCACTTGGCTGGCCCAGAGG	3MMs [5:6:8]	chr19
	OT4	GCCCTTCCTGGCTGGCCCAGTGG	4MMs [3:4:5:8]	chr4
	OT5	GCTGTTCCTGGCTGGCCCTGCAG	3MMs [5:8:19]	chr19
	OT6	GCTCACTGTGGCTGGCCCAGGAG	3MMs [4:6:7]	chr16
	OT7	TCTGCTACTGGCTGGCCCAGAGG	4MMs [1:5:7:8]	chr3
	OT8	CGTGGTGGTGGCTGGCCCAGCAG	4MMs [1:2:5:7]	chr17
sgR2	OT1	GTTTCAAAGGCCACAGTTAGCAG	3MMs [2:9:10]	chr8
	OT2	GTCTCAAAATACACAGTTAGCAG	3MMs [2:3:11]	chr18
	OT3	GCTTCAAAAACCACAGTAAGCAG	3MMs [3:8:12]	chr14
	OT4	GCCTCAAGATCTACAGTTAGTGG	4MMs [1:2:3:4]	chr9
	OT5	TGGACAAAATCCACAGTTAGGAG	4MMs [2:3:4:10]	chr19
	OT6	GGGACAAAACCCACAGTTAGTAG	3MMs [1:6:20]	chr1
	OT7	TCTTCGAAATCCACAGTTAAAGG	4MMs [1:3:7:10]	chrX
	OT8	TCATCAGAAACCACAGTTAGGGG	4MMs [2:3:7:10]	chr8
	OT9	GGGTCACAAACCACAGTTAGTAG	4MMs [1:3:5:9]	chr9

Table S3 | Summary of Embryo Microinjection of Cas9 mRNA ands gRNA.

gRNA	Survived/injected (%)	Blastocyst /Survived (%)	*Mstn* KO/ Blastocyst (%)
sgR1	11/14 (78%)	8/11 (73%)	6/8 (75%)
sgR2	8/10 (80%)	6/8 (75%)	4/6 (67%)

Table S4 | Oligonucleotides used in this study.

Oligonucleotides used for pX330-gRNA construction.

gRNA	Direction	Oligoes(5'→3')
sgR1	F	caccGCTGATCGTGGCTGGCCCAG
	R	aaacCTGGGCCAGCCACGATCAGC
sgR2	F	caccGCTTCAAAATCCACAGTTAG
	R	aaacCTAACTGTGGATTTTGAAGC
sgG	F	caccgAAGGGCCGGCTGAACCTTTG
	R	aaacCAAAGGTTCAGCCGGCCCTTc

Oligonucleotides used for PCR and T7E1 assay.

Primer sets	Direction	Primers (5'→3')	Products length (bp)
T7_sgG	F	TGCGGTAGGAGAGTGTTTGG	745
	R	CACCAGAAGACAAGGAGAATTGC	
T7_sgR1	F	TGTGGAGCAAGAGCCAATCA	571
	R	TTGTTTCCGTCGTAGCGTGA	
T7_sgR2	F	TTCCTCTGCCTCTCCTTCCA	694
	R	CACCCACAGCGGTCTACTAC	

Oligonucleotides used for detection of long range deletion of rabbit *Mstn*.

Primer set		Primers (5'→3')	Product length (bp)
LD	F	TGTGGAGCAAGAGCCAATCA	≈540
	R	TGGTCAAGGTACGAGAGGAC	

Oligonucleotides used for qRT-PCR

Gene	Direction	Primers (5'→3')	Gene Bank ID	Product Length (bp)
Myogenin	F	TGTGTAAGAGGAAGTCAGTGTCCA	NM_001177749.1	92
	R	CTCGAAGGCCTCGTTCACTTT		
β-actin	F	ATGCAGAAGGAGATCACCGC	NM_001101683.1	148
	R	ACTCCTGCTTGCTGATCCAC		

Oligonucleotides used for making template for in vitro transcription.

IVT-primers	Sequences (5'→3')
IVT_Cas9_F	taatacgactcactatagggAGAATGGACTATAAGGACCACGAC
IVT_Cas9_R	GCGAGCTCTAGGAATTCTTAC
IVT_sgR1_F	ttaatacgactcactatagGCTGATCGTGGCTGGCCCAG
IVT_sgR2_F	ttaatacgactcactatagGCTTCAAAATCCACAGTTAG
IVT_sgG_F	ttaatacgactcactataggAAGGGCCGGCTGAACCTTTG
IVT_gRNA_R	AAAAGCACCGACTCGGTGCC

Oligonucleotides used for PCR and T7E1 assay of OTs of sgG, sgR1 and sgR2.

gRNA	OTs	Direction	Primers (5'→3')	Products Length (bp)
sgG	OT1	F	AGGTTGGGTTGTCCACCTTG	511
		R	TTCAAGCAGGGCGAACAGTA	
	OT2	F	AATCCTCAGGACCCTCTCCC	500
		R	AGGCTTCTAGGTAAGCTGGC	
	OT3	F	TGAGTGAAGCAGTTCGACCC	738
		R	ACGTGAATGTGGATCAGGGAC	
	OT4	F	AGGGAGGTTGCCTAGGAGAG	532
		R	ACCTTATTTGTGCTGCCCGA	
	OT5	F	GCTGTGGTTGGGTGTGTTTC	591
		R	GTGTCACAAAGCTGCCAAGG	
	OT6	F	GGGATGCACCAGGAACAAGA	507
		R	AGGGGCCCTTGACTGTATCT	
	OT7	F	GTGGCCAGTTGCAATTTAGGG	665
		R	ACCTCTCCTGCAAACAGCTC	
	OT8	F	ACACCCAGACAGATCCTGGT	512
		R	AGTTCTGAGCAGTGCGAGAG	
	OT9	F	AGGTTCCTTGCAGAGATGCC	605
		R	GCCTGCTTAGAGGACTGACC	
	OT10	F	GGTCTGAAGGCAGAAGGAGC	576
		R	AGGCTCATGGAGGTGCTTTC	
sgR1	OT1	F	AGGATATGGGCGTCCTGAGA	671
		R	TCAGCAGGCTCTGTTCCTTG	
	OT2	F	GTAAAGGCAACAGCACTGCC	600
		R	GGTACCTACCCATCCCTGGT	
	OT3	F	GAGAGGAAACACATGGGCGA	635
		R	GCATCTACACTGCCACACCT	
	OT4	F	GGTTTCTGAGCAAGTGGGGA	615
		R	ACCCATCCTAACAACTGCCG	
	OT5	F	CACTACCTGCTATGCCGCAG	671
		R	GCACAGGGAGTTAGGAGCAG	

	OT6	F	ACGAGGGTCCAGCTATTCCT	670
		R	ACCCATCCTAACAACTGCCG	
	OT7	F	AAATGGACAGGGAGCTGTGG	482
		R	CTCTCCCTGCAACCTTAGGC	
	OT8	F	TTTCCTTGGCGGAGGATGAC	513
		R	GGGTCCTAGCTTGGGTTTCC	
sgR2	OT1	F	TCATTGCAGCTCAATTCACA	600
		R	TTTACCCACTACGCCAGAGC	
	OT2	F	CCAGTAAATATCTTTCAGCCTTTT	501
		R	TGCCAAAGGGAAACCCCCAT	
	OT3	F	GACTGGCTGCCGAAGTTTTC	716
		R	TGGCTGATTCTGTGGCTACC	
	OT4	F	GACCACCAGGCTAACACCAA	506
		R	GTGGTCAGGTACAACAGGGG	
	OT5	F	AGAAAAGCCTCAGTTCGGCA	473
		R	TCCCCACTTACTTTCTGGCG	
	OT6	F	CCCTCCATTCCAGCTTCCTG	552
		R	GCGAATAATCTGCCTGGCCT	
	OT7	F	GAGGGGGTCTTCAAAAAGTTG	415
		R	GATTGGGTTGGTTTGGATTG	
	OT8	F	TAGGGCGCCATTTTTCCCAT	534
		R	GGTCTGGGTTCATGGCTACC	
	OT9	F	AAGTGGATTGTTTTGATTTCTACA	435
		R	TAAGACCACTGCAAGCAGCA	

Table S5 | Schedule of superovulation of donor rabbits and goat

Donor Rabbits	Day	1		2		3		4		5				
	Injection Time	8:00	20:00	8:00	20:00	8:00	20:00	8:00	20:00	8:00	16:00			
	FSH (IU)	-	20	20	10	10	7.5	7.5						
	hCG (IU)								100					
	Mating								√					
	Embryo collection										√			
Donor Goat	Day	1~8		9		10		11		12		13		14
	Injection Time	8:00	20:00	8:00	20:00	8:00	20:00	8:00	20:00	8:00	20:00	8:00	20:00	8:00
	Sponge	√												
	FSH (IU)			50	50	25	25	25	25					
	PG (IU)							100						
	GnRH (IU)										100			
	Mating										√	√		
	Embryo collection													√

Table S6 | Summary of genome editing and tongue type and health status.

Recipient	Fetus	Genome editing efficiency*[1]			Tongue type*[2]	Heath status*[3]
		p1	*p2*	*p*		
FR1	#1	0.00	0.00	0.00	Normal	Partially eaten
FR2	#2	0.00	0.12	11.76	Normal	Partially eaten
FR5	#3	0.71	1.00	100.00	Enlarged	Stillborn
FR8	#4	0.88	0.78	97.22	Enlarged	Stillborn
	#5	0.00	0.56	55.56	Enlarged	Weak and dead
FR10	AF	0.00	0.00	0.00	-	Aborted
SR1	#1-1	1.00	0.50	100.00	Enlarged	Stillborn
	#1-2	1.00	0.00	100.00	Enlarged	Weak and dead
	#1-3	0.00	0.00	0.00	Normal	Alive
	#1-4	0.25	0.50	62.50	Enlarged	Weak and dead
SR2	#2-1	0.00	0.50	50.00	Normal	Weak and euthanized
	#2-2	0.00	0.00	0.00	Normal	Alive
	#2-3	0.00	0.86	85.71	Enlarged	Weak and dead
	#2-4	0.50	0.67	83.33	Normal	Weak and euthanized
	#2-5	0.88	0.40	92.50	Enlarged	Alive
	#2-6	0.56	0.90	95.56	Normal	Weak and dead
	#2-7	0.78	0.40	86.67	Enlarged	Weak and euthanized
SR5	#5-1	0.30	0.00	30.00	Normal	Alive
	#5-2	0.20	0.00	20.00	Normal	Alive
	#5-3	0.00	0.00	0.00	Normal	Alive
	#5-4	0.00	0.11	11.11	Normal	Alive
	#5-5	0.00	0.00	0.00	Normal	Alive
	#5-6	0.00	0.00	0.00	Normal	Dead due to illness
	#5-7	0.00	0.00	0.00	Normal	Alive
	#5-8	0.75	0.20	80.00	Enlarged	Alive
SR11	#11-1	0.89	0.80	97.78	Enlarged	Stillborn
SR12	#12-1	0.71	0.00	71.43	Enlarged	Alive
	#12-2	0.63	0.78	91.67	Enlarged	Alive
SR14	#14-1	0.63	0.67	87.50	Normal	Alive
	#14-2	0.00	0.00	0.00	Normal	Alive
	#14-3	0.00	0.11	11.11	Normal	Alive
	#14-4	0.00	0.00	0.00	Normal	Alive
	#14-5	0.43	0.00	42.86	Normal	Alive
S16	#16-1	1.00	0.00	100.00	Normal	Alive
	#16-2	0.50	1.00	100.00	Enlarged	Stillborn

*[1]: *p1* and *p2*, genome editing efficiency at sgR1 and sgR2 site respectively, were calculated by KO TA clones/clones sequenced; total genome editing efficiency *p* was calculate as (1-(1 - p1) (1 - p2)) * 100;

*[2]: Tongue status includes normal tongue and enlarged tongue;

*[3]: Health condition indicated stillborn, weak and dead, weak and euthanized, and alive. #5-6 died due to illness with the character of spotted lung and grey liver.
